# Four meta-analyses across 164 studies on atypical footedness prevalence and its relation to handedness

**DOI:** 10.1038/s41598-020-71478-w

**Published:** 2020-09-02

**Authors:** Julian Packheiser, Judith Schmitz, Gesa Berretz, David P. Carey, Silvia Paracchini, Marietta Papadatou-Pastou, Sebastian Ocklenburg

**Affiliations:** 1 Institute of Cognitive Neuroscience, Biopsychology, Department of Psychology, Ruhr University Bochum, Universitätsstraße 150, 44780 Bochum, Germany; 2 School of Medicine, University of St Andrews, St Andrews, UK; 3 Perception, Action and Memory Research Group, School of Psychology, Bangor University, Bangor, UK; 4 School of Education, Department of Primary Education, National and Kapodistrian University of Athens, Athens, Greece; 5 Department of Psychology, University of Duisburg-Essen, Essen, Germany

**Keywords:** Psychology, Human behaviour

## Abstract

Human lateral preferences, such as handedness and footedness, have interested researchers for decades due to their pronounced asymmetries at the population level. While there are good estimates on the prevalence of handedness in the population, there is no large-scale estimation on the prevalence of footedness. Furthermore, the relationship between footedness and handedness still remains elusive. Here, we conducted meta-analyses with four different classification systems for footedness on 145,135 individuals across 164 studies including new data from the ALSPAC cohort. The study aimed to determine a reliable point estimate of footedness, to study the association between footedness and handedness, and to investigate moderating factors influencing footedness. We showed that the prevalence of atypical footedness ranges between 12.10% using the most conservative criterion of left-footedness to 23.7% including all left- and mixed-footers as a single non-right category. As many as 60.1% of left-handers were left-footed whereas only 3.2% of right-handers were left-footed. Males were 4.1% more often non-right-footed compared to females. Individuals with psychiatric and neurodevelopmental disorders exhibited a higher prevalence of non-right-footedness. Furthermore, the presence of mixed-footedness was higher in children compared to adults and left-footedness was increased in athletes compared to the general population. Finally, we showed that footedness is only marginally influenced by cultural and social factors, which play a crucial role in the determination of handedness. Overall, this study provides new and useful reference data for laterality research. Furthermore, the data suggest that footedness is a valuable phenotype for the study of lateral motor biases, its underlying genetics and neurodevelopment.

## Introduction

A consistent behavioural phenomenon in many species is that most prefer one limb over the other^1–3^. Most laterality research in humans has studied the prevalence and origins of handedness as it has been used as a proxy for asymmetrical cerebral organization^4–8^. However, humans demonstrate another highly established limb preference, namely to favour one foot over the other^9,10^. However, to this day, a precise estimate on the prevalence of atypical footedness in the population is still missing

Footedness has been suggested to be an on a par or even better predictor than handedness for the lateralization of language in the brain. Searleman^11^ for example assessed language lateralization in 373 participants via a dichotic listening task and found that footedness was the single best predictor. In a large-scale study investigating 1554 individuals, footedness was also a better predictor of language lateralization as compared to handedness on the categorical level^12^. These results were supported by other studies and could be extended to emotional lateralization as well^13–15^. The reason for this increased association with these phenotypes might be linked to the “purity” of the measure. Footedness is most likely less influenced by cultural norms or social teaching compared to handedness for which influences of these factors have been documented repeatedly^16–19^. For that reason, footedness might be a better suited candidate phenotype to approximate brain lateralization in large cohorts.

Another important reason to study footedness relates to the precise relationship between different lateral motor biases. It still remains rather unclear to what extent footedness and handedness are associated. Recently, the prevalence of handedness has been investigated in a large-scale meta-analysis and it was identified that 10.60% of the population are left-handed^20^. However, there is much uncertainty whether the extent of left-footedness follows a similar distribution. Illuminating the relationship between handedness and footedness could potentially give insights for the design of studies investigating the genetic basis of motor lateralization. Several studies have investigated the relationship between handedness and footedness and found a significant positive correlation suggesting that there is a shared underlying biological mechanism giving rise to lateralized motor functions^14,21–26^. However, the correlations found in these studies differ vastly ranging from 0.2 to 0.8 indicating that individual studies are unlikely to provide reliable estimates about this relationship in the population.

A major problem in providing a precise prevalence of atypical footedness and finding a reliable estimate on the association with handedness stems from methodological problems in laterality research. First, lateral motor preferences are skewed within the population with right-sided preferences being more prevalent than left-sided and mixed preferences^27^. If a random sample is drawn from the population, this skewedness then requires a very large sample size in order to represent each group with sufficient power^25^. Furthermore, due to a large variety of classification systems and assessment batteries, it is difficult to compare results from individual studies even if they comprised large sample sizes. For example, Tran and Voracek^28^ found that in a substantial sample of 12,720 participants, there were 1,043 (8.2%) left-footers and 3,839 (30.2%) mixed-footers. Martin and Porac^29^ sampled another large cohort of 3,716 participants and found a substantially larger proportion of left-footed individuals (560 or 15.1%), but almost no mixed-footers (68 or 1.8%). As evident from these results, individual samples can hardly achieve a representative prevalence of the population due to potential sampling biases and different measurements of footedness. Furthermore, both the fields of psychology and neuroscience have suffered from a massive replication crisis due to underpowered studies reporting spurious effects, publication bias, and *p*-hacking^30,31^. One solution to these issues is the application of large-scale meta-analyses as they can provide meaningful insights into the true point estimates in the population, are more resistant to sampling errors, and can identify publication biases. They additionally allow for the investigation of moderating factors that potentially influence foot preference^32^.

### Potential moderators of the prevalence of footedness

There is a wide body of literature claiming that individuals with psychiatric and neurodevelopmental disorders exhibit elevated rates of non-right-handedness, for example in schizophrenia^33^, autism spectrum disorders (ASD;^34^), dyslexia^35^, intellectual disability^36^, but also post-traumatic stress disorder (PTSD) and depression^37,38^. It is thus conceivable that there is a similar relation with footedness as these lateral biases are significantly associated. A higher prevalence of atypical footedness has been found in individuals with schizophrenia spectrum disorders^39–41^. In a large sample of 2,800 participants, it was even shown that mixed-footedness is a better predictor than mixed-handedness for schizotypical traits, potentially due to the reduced cultural influences on the measure^26^. In neurodevelopmental disorders, such as dyslexia and attention deficit hyperactive disorder (ADHD), footedness has been found to be significantly more atypical, i.e. less right-lateralized, compared to healthy controls^42–44^. Studies investigating the relationship between footedness and ASD found no association^45,46^. However, these studies were severely underpowered with sample sizes as low as 12 individuals calling into question whether they had the means to identify any effects. In conclusion, there is evidence from individual studies that footedness might be linked to similar clinical disorders as is the case for handedness.

Footedness also represents an important topic in the context of sports. In symmetrical sports like football, ambilaterality is considered advantageous as there is equal opportunity to use either limb facilitating competitive play at the highest level^47–49^. Furthermore, players with ambilateral skill are more polyvalent in their position on the field as they can be used on either side. Grouios and colleagues^50^ investigated the prevalence of mixed-footedness in 1,509 football players and indeed found higher rates of mixed-footedness in professional (45.9%) and semi-professional (30.3%) soccer players compared to amateurs (12.6%) or non-sporting university students (9.1%). Interestingly, a study investigating the salary returns of players in the German Bundesliga in relation to their two-footed ability found substantial evidence of higher premiums if players demonstrated ambilateral qualities, even after controlling for other performance scores^51^.

For handedness, not only ambilaterality, but also a left preference has been proposed to provide an advantage in sports as left-sided athletes are encountered less often during practice and therefore opponents will have less experience in playing against them^52,53^. Carey et al.^54^ investigated both the incidence of left-footedness as well as the relative success rate of left- compared to right-footers in professional football players during the 1998 World Cup in France. They found neither evidence of increased rates of left-footedness compared to the general population, nor that left-footed players were overall more successful in their actions. However, while some studies replicated these findings on the prevalence of left-footedness in football players^55^, Verbeek and colleagues^56^ found a ratio of 30.9% left-footers amongst 497 football players. Overall, the findings seem to suggest that mixed-footedness is observed more often in football players compared to the general population whereas findings regarding an increased rate of left-footedness remain rather inconclusive.

Another important and to this day highly debated question is whether motor asymmetries are present from early gestation and stable across the entire lifetime^2^. For both handedness and footedness, cross-sectional studies have suggested that right-sided preferences in the population increase with age^9,57–59^. Several studies investigating the prevalence of atypical footedness during childhood have reported increased rates of specifically mixed-footedness compared to adult samples whereas the prevalence of left-footedness seemed to be similar in child and adult samples^9,60,61^. A possible explanation for this phenomenon has been proposed by theories of differential hemispheric aging. The right hemi-aging model assumes that the right hemisphere is prone to a quicker functional decline than the left hemisphere^62,63^ possibly leading to increased left-hemispheric dominance as humans grow older. It has to be noted however that this model is mostly supported when comparing younger to older adults leaving open whether this model also applies to individual development during childhood and adolescence. A non-biological explanation for this phenomenon is also possible since the assessment of footedness in children has often been performed using single or very few motor tasks like kicking a ball or hopping on one foot^23,64,65^. Thus, the increased prevalence of mixed-footedness might be a result of less accurate test batteries that do not allow for a more specific determination of footedness as is common in adult footedness research. For handedness, it has been suggested that age effects are rather due to generational effects instead of changes in limb preference across the individual lifetime^66–68^. However, as there is little evidence for social pressure against left-foot preference, this type of explanation might be a less likely account for possible age effects on the prevalence of left-footedness.

### Aims of the present study

The first goal of the present meta-analytic study was to provide an accurate and reliable point estimate of atypical footedness in the population. For that purpose, we conducted four meta-analyses using different classification schemes ranging from a conservative trichotomous left-mixed-right classification to a liberal dichotomous non-right/right classification. We then investigated sex differences in footedness since males have been demonstrated to be more often left-handed compared to females^69^. Furthermore, we investigated the relationship between footedness and handedness in the population by specifically analysing those studies that determined both upper and lower limb preferences. We additionally investigated moderating factors on the prevalence of atypical footedness, namely (1) whether or not samples came from a population diagnosed with a psychiatric or neurodevelopmental disorder, (2) whether sampling was conducted in experienced athletes or in a non-sporting population and (3) whether samples consisted of children or adult participants. Furthermore, the test instrument during footedness assessment was used as a moderator since the type of measurement used has been demonstrated to influence the proportion of left-handedness^20^.

## Methods

### New data

This study includes new data on footedness from the Avon Longitudinal Study of Parents and Children (ALSPAC) study, a UK population-based longitudinal cohort. Pregnant women resident in the county of Avon, UK, with expected dates of delivery 1st April 1991 to 31st December 1992 were invited to take part in the study, resulting in 14,062 live births and 13,988 children who were alive at 1 year of age^70,71^.

Footedness was assessed using 4 items (kick a ball, pick up a pebble with the toes, stamp on something, climb a step) rated on a 3-point scale (left, either, right) in both mothers and children. In children, footedness was assessed at 42 months based on maternal report. In mothers, the same items were assessed using self-report. In order to develop a score where the lowest values indicate left-footedness and the highest values indicate right-footedness, “left”-responses were coded as 1, “either”-responses were coded as 2 and “right”-responses were coded as 3 and a mean value was calculated from the number of valid responses. Finally, a summary variable was created by recoding this variable as follows: children: < 2.00: left, >  = 2.00 and =  < 2.50: mixed, > 2.50: right; mothers: < 1.75: left, >  = 1.75 and =  < 2.50: mixed, > 2.50: right.

Similarly, handedness was assessed via maternal report in children at 42 months and self-report in mothers. Handedness assessment was based on six items (drawing/writing, throwing, colouring, holding a toothbrush, cutting, hitting) and a summary score was obtained: children: < 2.00: left, >  = 2.00 and =  < 2.75: mixed, > 2.75: right; mothers: < 1.50: left, >  = 1.50 and =  < 2.60: mixed, > 2.60: right.

Ethical approval for the study was obtained from the ALSPAC Ethics and Law Committee and the Local Research Ethics Committees. Informed consent for the use of data collected via questionnaires and clinics was obtained from participants following the recommendations of the ALSPAC Ethics and Law Committee at the time. Please note that the ALSPAC study website contains details of all the data that is available through a fully searchable data dictionary and variable search tool (https://www.bristol.ac.uk/alspac/researchers/our-data/).

Footedness data from the ALPSAC cohort were available for 9,559 mothers. By self-report, 29 individuals reported current or previous drug addiction, 75 reported current or previous alcoholism, 10 reported previous schizophrenia, 189 reported current or previous anorexia nervosa, 749 reported current or previous severe depression, and 205 reported other psychiatric problems. A total of 1,031 mothers were thus assigned to the clinical group (age: M = 32.28, SD = 5.12) (diagnoses were not mutually exclusive). The remainder of 8,528 mothers not suffering from any of these conditions were assigned to the control group (age: M = 32.01, SD = 4.66).

Footedness data were available for 9,817 children. Of these, 60 were excluded because of reported physical disabilities since this might influence the foot preference. Psychiatric assessment was conducted using the parent version of the Development and Well-being Assessment (DAWBA^72^) when the children were 7.5 years old. The DAWBA is a diagnostic tool based on DSM IV criteria for a number of psychiatric disorders (American Psychiatric Association, 1994). Overall, 140 children had a diagnosis of ADHD (either inattentive, hyperactive-impulsive, or combined), 221 had a diagnosis of oppositional-conduct disorder (oppositional defiant disorder, conduct disorder, disruptive behaviour disorder not otherwise specified (NOS)), 217 had a diagnosis of any anxiety disorder (separation anxiety disorder, specific phobia, social phobia, PTSD, OCD, generalized anxiety disorder, anxiety disorder NOS), and 36 had a diagnosis of a depressive disorder (major depressive disorder, depressive disorder NOS) and were assigned to the clinical sample. This sample consisted of 485 children (as diagnoses were not mutually exclusive) (326 females, age: M = 3.55, SD = 0.07; age is reported for the time of footedness assessment). Due to reports of elevated levels of atypical behavioural asymmetries in dyslexia^35^, we also included a dyslexia sample. After exclusion of the clinical sample, an assignment of dyslexia was based on a performance IQ of >  = 85 at the age of 8.5, an age-adjusted single word reading score  ≤ − 1 SD for both a 40-word reading test at 7.5 years and a 12-word reading test at 9.5 years^73^. The dyslexia sample consisted of 268 children (167 females, age: M = 3.55, SD = 0.06). Children free from physical disabilities, psychiatric diagnosis and not included in the dyslexia sample were assigned to the control sample (n = 9,004; 4,557 females, age: M = 3.56, SD = 0.08). Footedness data for all subgroups in the ALSPAC cohort are included in Supplementary Table 1. Using individual data points, we calculated Spearman rank correlations between footedness and handedness in all subgroups of the ALSPAC cohort. There was strong evidence in all subgroups that footedness and handedness were significantly correlated (all *p*’s < 0.001; *r* = 0.50 for children in the control group; *r* = 0.55 for children in the clinical group; *r* = 0.52 for children in the dyslexic group; *r* = 0.48 for mothers in the control group; *r* = 0.53 for mothers in the clinical group). 95% confidence intervals did overlap between all groups, suggesting no group differences in the strength of correlation.

### Study selection for the meta-analysis

Study selection and conduction of meta-analyses followed the PRISMA guidelines^74^. The aim of the PRISMA guidelines is to improve the transparency of reporting systematic reviews and meta-analyses via a 27-item checklist that decouples several items that were used in previous checklists (e.g., the QUOROM checklist^75^). The following procedure was used for the selection and inclusion of research studies into the present meta-analyses: in a first step, the electronic databases PubMed (https://www.ncbi.nlm.nih.gov/pubmed/), Web of Science (https://www.webofknowledge.com), and Google Scholar (https://scholar.google.de/) were searched for the terms “footedness”, “foot use” or “feet use”, “foot preference” or “feet preference”, “foot asymmetry” or “feet asymmetry”, “lower limb preference” or “lower limb asymmetry”. All reference lists of eligible publications were then inspected for other research items associated with footedness. Furthermore, review articles on human footedness were inspected^9,76^ to identify articles that could not be found in the above mentioned electronic databases. E-mail requests were sent to corresponding authors if (1) articles could not be retrieved from online databases and (2) if the study clearly stated that participants’ footedness was measured, but did not report footedness in a manner to extract the data properly. Data collection was conducted by JP, JS and GB and concluded in February 2020. Data extraction was conducted by JP and JS. Cohen’s Kappa was used to determine interrater reliability as it can account for chance based agreement^77^. The interrater reliability between JP and JS was at κ = 0.88 which is considered a strong level of agreement in accordance with McHugh^78^. Disagreements were resolved by discussion.

### Inclusion and exclusion criteria

This study used the following criteria to determine in- or exclusion of datasets:

1. Participants: Only physically healthy participants were used to control for potential influences of physical disabilities on foot preference. Samples including participants diagnosed with conditions such as stroke^79^, arthrosis^80^, or trisomy^81^ were excluded from analysis. We specifically included patients with neurodevelopmental or psychiatric disorders (e.g., ASD^45^, schizophrenia^39^, or dyslexia^44^) and their control groups. Control groups were treated as healthy cohorts for all analyses.
2. Age: Participants of all ages were included into the study. Datasets were classified as children cohorts if the participants’ age ranged between 0 and 17 years and classified as adult cohorts if the participants’ age was 18 years or above. If the cohort consisted both of children and adults (*n* = 21 studies), they were excluded from the moderator analysis comparing children and adult cohorts.
3. Publication language: All articles included in the present meta-analysis were written in English.
4. Footedness report: Data from studies was only included if a clear footedness prevalence was reported. Thus, studies reporting only lateralization quotients or *p*-values for footedness were excluded. The footedness measure had to be reported either in a left/right, left/mixed/right, or non-right/right classification system for further inclusion. A single study reported their data in a left/non-left classification and was therefore excluded^82^.
5. Pre-selection of the sample: Studies were excluded if the sample was pre-selected to solely contain left- or right-footed individuals^83–85^ or purposely balanced the number of left- and right-footed participants in the sample^10,14,86^. If studies did not explicitly mention a pre-selection for footedness, but were suspicious due to the absence of non-right-footed individuals, the authors chose to exclude those studies as well^87,88^. This was the case if the study featured more than 20 individuals without any left- or mixed-footers as this would have been very unlikely in a randomly drawn sample.

**Figure 1:**
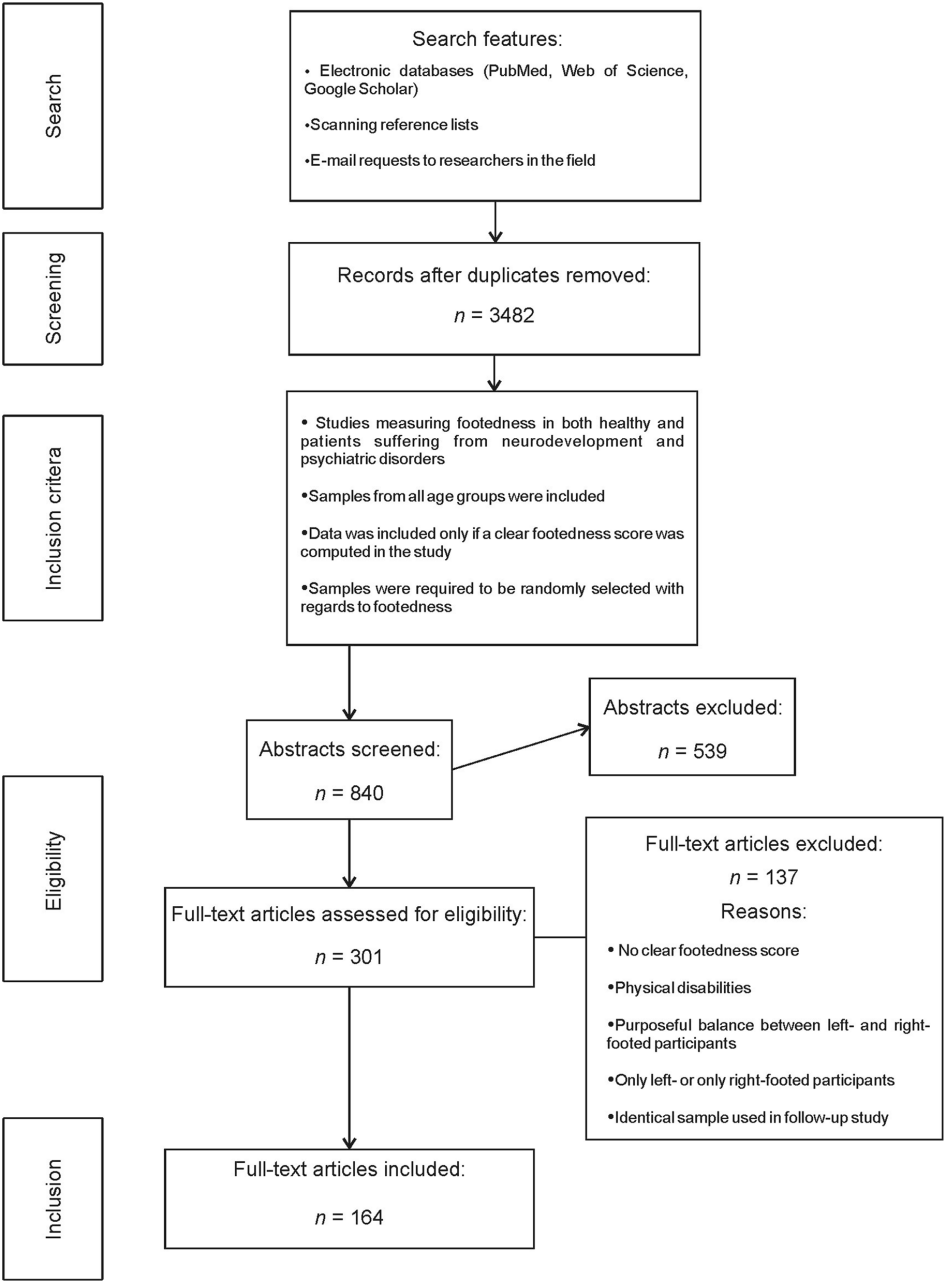
Flow diagram for search and inclusion criteria in the meta-analysis in accordance with the PRISMA guidelines for systematic reviews and meta-analyses^74^. A complete list of studies included in the meta-analysis can be found in Supplementary Table S1.

As mentioned under (4), the present meta-analysis included studies using Left–Right (L–R), Left-Mixed-Right (L-M-R), and non-Right–Right (nonR–R) classification systems. If studies used an even more precise classification system like strong left- and right-footedness, weak left- and right-footedness, and no preferences, these were converted to L-M-R with weak and strong being classified into one category. For age groups, datasets of single studies were separated if a clear distinction of age between the datasets could be identified. In total, 217 datasets from 164 studies including new data from the ALSPAC cohort totalling *n* = 145,150 individuals were included in this meta-analysis (see Fig. 1).

### Moderator analyses and variables

1. Sex: We investigated the moderating effects of sex when data was separately reported for males and females.
2. Handedness: We investigated the moderating effects of handedness when data was separately reported for left- and right-handers (or left-, mixed, and right-handers).
3. Clinical sample: Datasets were divided into healthy cohorts (198 datasets) including control groups from patient studies and clinical cohorts (13 datasets) including patients suffering from various psychiatric and neurodevelopmental disorders.
4. Sporting ability: Datasets were classified into experienced athletes (32 datasets) and amateurs (179 datasets). To be classified as an experienced athlete, studies either explicitly stated so or were classified by the authors based on at least 4 years of experience in the respective sport.
5. Age group: Datasets were classified into children (59 datasets) and adult cohorts (137 datasets).
6. Mean age: when available, age was included as a continuous meta-regressor.
7. Instrument: The assessment instrument for measuring footedness was classified into four categories and then used as a moderator variable. We chose specifically the Waterloo Footedness Questionnaire (WFQ^15^; 62 datasets), the Lateral Preference Inventory (LPI^27^; 22 datasets), and kicking a ball (39 datasets) as the first three categories since they were used in the large majority of cases. The fourth category contained other preference inventories used for assessing footedness and was therefore termed “other” (71 datasets).
8. Response format: Responses were either binary (left–right; 73 datasets), graded on a 3-point scale (left—mixed—right; 59 datasets), or on a 5-point scale (always left—mostly left—mixed—mostly right—always right; 69 datasets).
9. Study location: Location of the study was used as a moderator to identify cultural influences on foot preference. The affiliation of the leading author was used to determine study location unless it was otherwise specified. Locations were grouped into the following regions: Europe (94 datasets), America (USA, Canada, South America) (63 datasets), Africa (6 datasets), Asia (40 datasets), and New Zealand and Australia (12 datasets).
10. Year of publication: We included the publication year as a continuous meta-regressor. Since very early studies have demonstrated to significantly influence the prevalence of handedness, i.e. studies from before 1940^89,90^, year of publication was also analysed as a categorical variable with the five groupings (a) < 1976, (b) 1976–1985, (c) 1986–1995, (d) 1996–2007, and (e) 2008–2019 in accordance with the handedness meta-analysis by Papadatou-Pastou et al.^20^.

Moderator analyses were mainly conducted using the nonR–R classification system as this analysis contained all datasets since it was the most inclusive (all datasets could be converted to nonR–R). However, we specifically conducted moderator analyses in the L-M-R classification for sporting ability, age group, and instrument as the literature has suggested specific effects for left- and mixed-footedness.

### Statistical analysis

Meta-analysis was performed using the R package robumeta^91^. We performed four separate meta-analyses using random effects models, according to the classification scheme of the studies. For all hypothesized effects, we used an alpha level of 5%. For all exploratory effects, we used a Bonferroni corrected threshold to control for multiple comparisons due to the large number of individual tests performed. The following groups were used for analysis:

1. Non-right-footedness (when assessed as a non-right/right (nonR–R) classification).
2. Left-footedness (when assessed as a left–right (L–R) classification).
3. Left-footedness (when assessed as a left-mixed-right (L-M-R) classification).
4. Mixed-footedness (when assessed as a left-mixed-right (L-M-R) classification).

The following analysis steps were carried out for each group:

- Step 1: Footedness prevalence was calculated by dividing the number of left-, mixed-, or non-right-footed participants by sample size for each dataset. Prevalence is reported with corresponding 95% confidence intervals.
- Step 2: Meta-regression was performed using correlated robust variance estimation (RVE) models.
- Step 3: Each grouping was tested for homogeneity using the *I*^2^ index reflecting the variance explained by heterogeneity across studies. The *I*^2^ index levels can be described as low, moderate, and high, when they are close to 25%, 50%, and 75%, respectively^92^. Additionally, the Tau^2^ index was computed to specify if there was variance between studies.
- Step 4: Sex and handedness influences were investigated for each of the four classification schemes. Footedness prevalence was compared between sexes and between handedness groups using the *t*-statistic (and corresponding *p*-values). Very few studies provided footedness broken down by sex and handedness, thus we could not test for an interaction between these two moderators.
- Step 5: The funnel plot (funnel() function) was visually inspected to identify small study bias. Furthermore, we used Egger’s regression test (regtest() function) to provide a qualitative estimate of the asymmetry of the funnel plot. Finally, the trim and fill method (trimfill() function)^93^ of the R metafor package^94^ was used to make the funnel plot symmetrical by omitting and/or adding hypothetical data points due to an asymmetrical distribution. For this analysis, we included only published datasets.
- Step 6: Moderator analyses were conducted separately for all moderator variables other than handedness and sex on all data sets using the nonR–R classification. Handedness and sex were excluded here as they only comprised a subsample of the data. Interactions with sex and handedness were tested for all other moderators. As for all other conducted analyses, we applied a conservative Bonferroni correction if the specific effect was not hypothesized.

### Ethical approval

The study was approved by the ALSPAC executive committee at the University of Bristol (Proposal B3233). No further approval was required as no other new data was generated in our study.

## Results

### Meta-analysis

#### Non-right-footedness (nonRight–Right classification)

**Figure 2:**
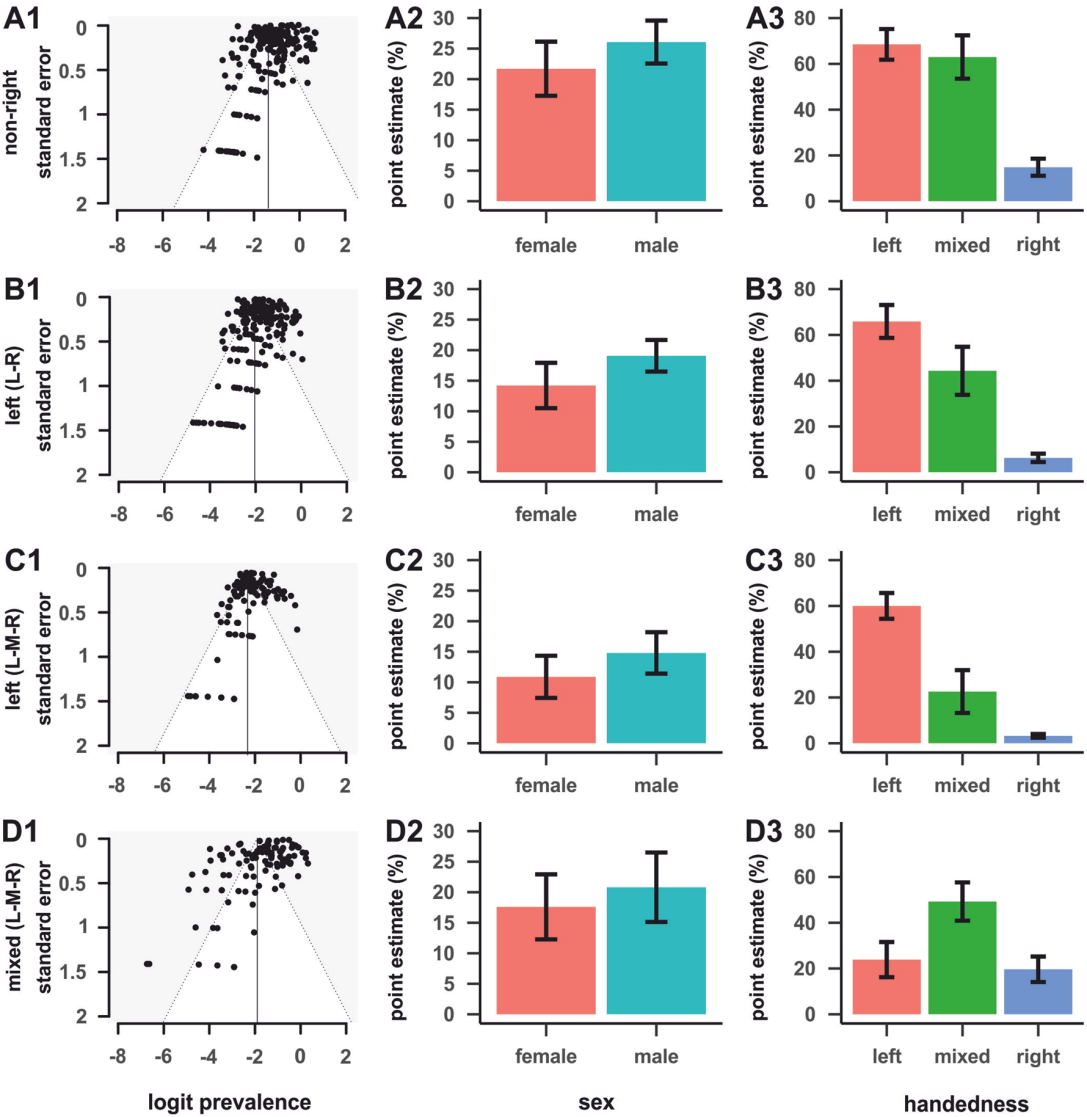
Funnel plots of standard errors on logit prevalence (1), sex averages in point estimates (2), and handedness averages in point estimates (3) for non-right-footedness (**A**), left-footedness (L–R classification) (**B**), left-footedness (L-M-R classification) (**C**), and mixed-footedness (L-M-R classification) (**D**). The left-sided asymmetries in the funnel plots indicate that there were more studies with high error rates when the logit prevalence was below average indicating that small samples were imprecise in their estimates. Error bars represent the 95% CI. Please note that the nonR–R classification system contains data from all studies whereas the other graphs only contain data when it could be converted to the relevant classification system.

A total of *k* = 217 datasets (from 164 studies including the ALSPAC cohort) were included in this meta-analysis, totalling *n* = 145,135 individuals. The first aim of the meta-analysis was to provide an accurate estimation on the prevalence of atypical footedness in the population. Simple meta-analysis using robumeta resulted in a point estimate of 23.70% for non-right-footedness with a 95% confidence interval (CI) = 21.40%, 25.90% (see SI Fig. 1 for a forest plot). Heterogeneity among the datasets was found to be high (*I*^2^ = 99.50%, Tau^2^ = 185.83). Egger’s regression test for funnel plot asymmetry revealed clear evidence for small study bias meaning that small studies showed a larger effect compared to large ones (*z* = − 5.89, *p* < 0.001). A visual inspection of the funnel plot revealed the same result (see Fig. 2A1). According to the trim and fill test, 17 studies (SE = 9.28) will need to be imputed to the right of the mean, corresponding to higher non-right-footedness rates in order for the funnel plot to be symmetrical.

We were further interested if sex differences between individuals affected the prevalence of atypical footedness and therefore repeated the analysis for datasets from which sex data could be extracted. For females, the point estimate was 21.70% (95% CI = 17.30%, 26.10%). For males, the point estimate was 26.10% (95% CI = 22.60%, 29.60%) (see Fig. 2A2). There was evidence that non-right-footedness prevalence was higher in males than in females (+ 4.38%, SE = 2.01%, 95% CI = 0.36%, 8.39%, *t*(73) = 2.17, *p* < 0.05). The same analysis was repeated for datasets containing handedness data to identify the relationship between footedness and handedness. Here, the point estimate was 14.80% (95% CI = 11.10%, 18.60%) for right-handers, 63.00% (95% CI = 53.50%, 72.50%) for mixed-handers, and 68.50% (95% CI = 61.80%, 75.20%) for left-handers (see Fig. 2A3). There was strong evidence for a higher prevalence in non-right-footedness in mixed-handers (+ 48.10%, SE = 4.91%, 95% CI = 38.30%, 58.00%, *t*(47) = 9.80, *p* < 0.001) and left-handers (+ 53.70%, SE = 3.61%, 95% CI = 46.40%, 60.90%, *t*(47) = 14.90, *p* < 0.001) as compared to right-handers. There was no evidence for a difference in non-right-footedness prevalence between left- and mixed-handers (*t*(40) = 1.07, *p* = 0.289).

### Left-footedness (left–right classification)

A total of *k* = 201 datasets (from 150 studies) were included in the analysis, totalling *n* = 121,302 individuals. Simple meta-analysis using robumeta gave an estimate of the left-footedness (L–R) prevalence of 16.10% with a 95% confidence interval (CI) = 14.40%, 17.70%. Heterogeneity among the datasets was found to be high (*I*^*2*^ = 96.42%, Tau^2^ = 43.14). Egger’s regression test for funnel plot asymmetry revealed clear evidence for small study bias (*z* = − 4.59, *p* < 0.001), as did the visual inspection of the funnel plot (see Fig. 2B1). However, according to the trim and fill test for random effects, no studies needed to be ‘trimmed’ or ‘filled’.

As for non-right-footedness, we repeated the analysis for data broken down by sex and handedness. For females, the point estimate was 14.20% (95% CI = 10.50%, 17.90%). For males, the point estimate was 19.10% (95% CI = 16.50%, 21.70%) (see Fig. 2B2). There was evidence that left-footedness (L–R) prevalence was higher in males than in females (+ 4.86%, SE = 1.83%, 95% CI = 1.21%, 8.52%, *t*(66) = 2.66, *p* < 0.01). Regarding handedness, the point estimate was 6.28% (95% CI = 4.45%, 8.11%) for right-handers, 44.30% (95% CI = 33.80%, 54.80%) for mixed-handers, and 65.87% (95% CI = 58.68%, 73.06%) for left-handers (see Fig. 2B3). There was strong evidence that the prevalence of left-footedness (L–R) was higher in mixed-handers (+ 38.02%, SE = 5.28%, 95% CI = 27.38%, 48.66%, *t*(45) = 7.20, *p* < 0.001) and left-handers (+ 59.59%, SE = 3.77%, 95% CI = 52.01%, 67.18%, *t*(45) = 15.82, *p* < 0.001) as compared to right-handers. There was evidence for a difference in left-footedness (L–R) prevalence between left- and mixed-handers (*t*(38) = 3.69, *p* < 0.001).

### Left-footedness (L-M-R)

A total of *k* = 109 datasets (from 81 studies) were included in the analysis, totalling *n* = 99,490 individuals. Simple meta-analysis using robumeta gave an estimate of a left-footedness (L-M-R) prevalence of 12.10% with a 95% confidence interval (CI) = 10.40%, 13.90%. Heterogeneity among the datasets was found to be high (*I*^2^ = 94.54%, Tau^2^ = 22.82). Egger’s regression test for funnel plot asymmetry revealed clear evidence for small study bias (*z* = − 3.73, *p* < 0.001), as did the visual inspection of the funnel plot (see Fig. 2C1). According to the trim and fill test, three studies (SE = 6.34) will need to be imputed to the right of the mean, corresponding to higher left-footedness (L-M-R) rates in order for the funnel plot to be symmetrical.

We again investigated sex effects on the prevalence of left-footedness in the L-M-R classification system. For females, the point estimate was 10.90% (95% CI = 7.42%, 14.30%). For males, the point estimate was 14.80% (95% CI = 11.41%, 18.20%) (see Fig. 2C2). There was evidence that left-footedness (L-M-R) prevalence was higher in males than in females (+ 3.92%, SE = 1.89%, 95% CI = 0.06%, 7.77%, *t*(33) = 2.07, *p* < 0.05). For handedness, the point estimate was 3.23% (95% CI = 2.41%, 4.05%) for right-handers, 22.60% (95% CI = 13.25%, 31.95%) for mixed-handers, and 60.01% (95% CI = 54.37%, 65.65%) for left-handers (see Fig. 2C3). There was strong evidence that the prevalence of left-footedness (L-M-R) was higher in mixed-handers (+ 19.37%, SE = 4.54%, 95% CI = 10.06%, 28.68%, *t*(27) = 4.27, *p* < 0.001) and left-handers (+ 56.78%, SE = 2.76%, 95% CI = 51.12%, 62.44%, *t*(27) = 20.57, *p* < 0.001) as compared to right-handers. There was evidence for a difference in left-footedness (L-M-R) prevalence between left- and mixed-handers (*t*(27) = 6.84, *p* < 0.001).

### Mixed-footedness (L-M-R)

A total of *k* = 109 datasets (from 81 studies) were included in the analysis, totalling *n* = 99,490 individuals. Simple meta-analysis using robumeta gave an estimate of the mixed-footedness prevalence of 20.20% with a 95% confidence interval (CI) = 17.00%, 23.40%. Heterogeneity among the datasets was found to be high (*I*^2^ = 99.20%, Tau^2^ = 101.77). Egger’s regression test for funnel plot asymmetry revealed clear evidence for small study bias (*z* = − 5.94, *p* < 0.001), as did the visual inspection of the funnel plot (see Fig. 2D1). According to the trim and fill test, 22 studies (SE = 6.86) will need to be imputed to the right of the mean, corresponding to higher mixed-footedness rates in order for the funnel plot to be symmetrical.

A final comparison for sex differences revealed that the point estimate for females was 17.60% (95% CI = 12.30%, 22.90%) and 20.80% for males (95% CI = 15.10%, 26.50%) (see Fig. 2D2). There was no evidence for a sex difference (*p* = 0.212). An effect of handedness could however be found as the point estimate was 19.70% (95% CI = 14.10%, 25.20%) for right-handers, 49.20% (95% CI = 40.90%, 57.60%) for mixed-handers, and 23.90% (95% CI = 16.20%, 31.60%) for left-handers (see Fig. 2D3). Here, there was strong evidence that the prevalence of mixed-footedness was higher in mixed-handers (+ 29.58%, SE = 4.30%, 95% CI = 20.75%, 38.40%, *t*(27) = 6.87, *p* < 0.001) as compared to right-handers, while there was no evidence for a difference between left- and right-handers (*p* = 0.219). There was evidence for a difference in mixed-footedness prevalence between left- and mixed-handers (*t*(27) = 5.31, *p* < 0.001).

In summary, we could determine that the point estimate of atypical footedness ranges from 12.1% using the most conservative classification system to 23.7% using the most liberal classification system. Males exhibited higher rates of left-footedness compared to females. Handedness also had strong effects on the prevalence of footedness with right-handers almost never being left-footed.

### Moderator variable analysis

Heterogeneity across all studies within each of the four meta-analyses was generally high indicating that footedness prevalence might be moderated by other variables. We therefore tested a variety of moderators that could potentially influence the present findings.

### Clinical samples

**Figure 3:**
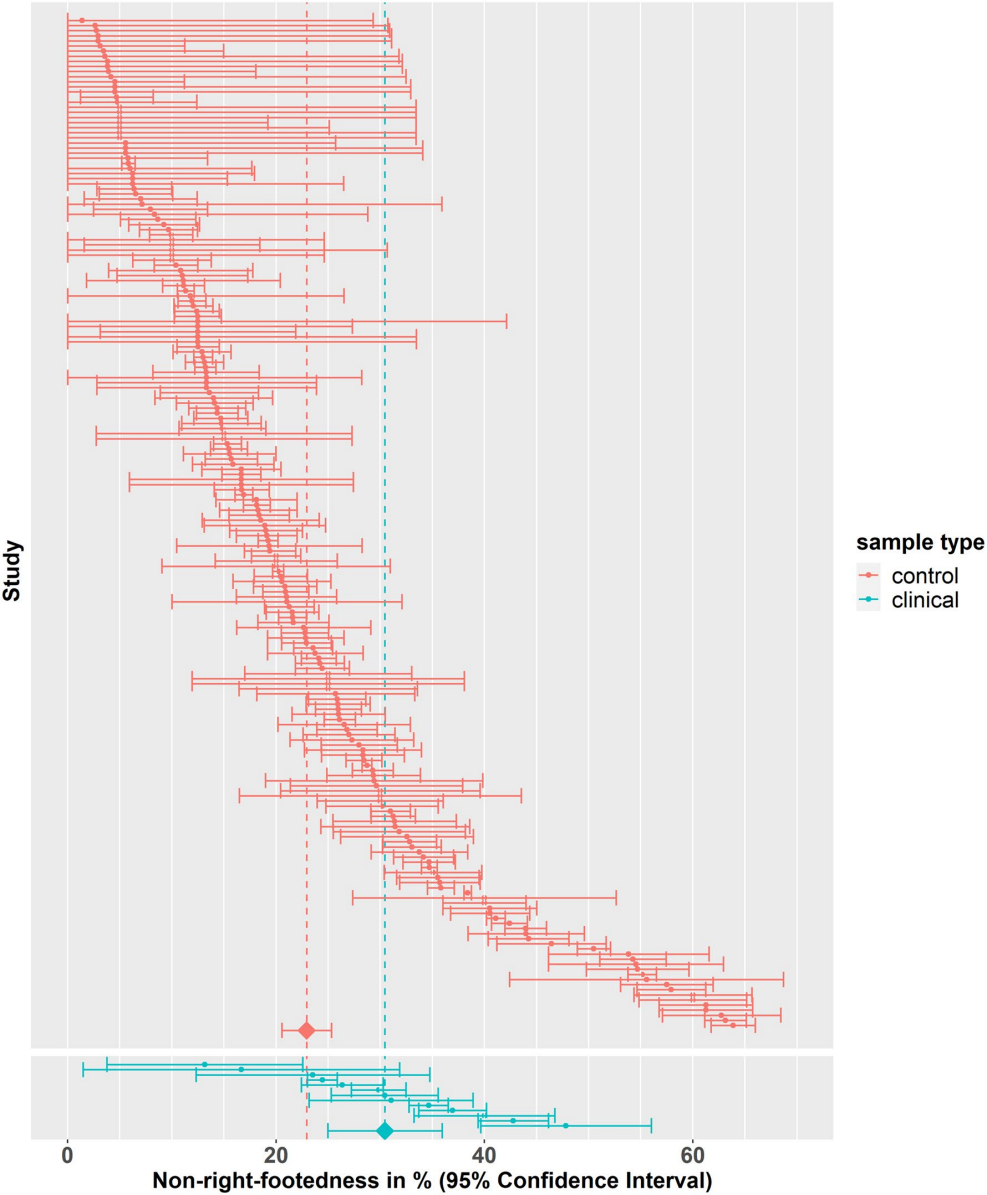
Forest plot for non-right-footedness grouped by sample type (control vs. clinical cohorts). The 95% confidence interval for each study is represented by a horizontal line and the point estimate is represented by a dot. The dashed lines and diamonds indicate the point estimates for the control (red) and the clinical (cyan) group.

We asked whether participants diagnosed with neurodevelopmental or psychiatric disorders demonstrated higher rates of atypical footedness. Sample type (control or clinical sample) was extracted from 211 datasets (158 studies). The prevalence of non-right-footedness was found to be 22.90% (95% CI 20.60%, 25.30%) in control samples (198 datasets) and 30.50% (95% CI 25.00%, 35.90%) in clinical samples (13 datasets) (see Fig. 3). There was evidence that the prevalence of non-right-footedness was higher in clinical samples (+ 7.51%, SE = 3.06%, 95% CI = 1.46%, 13.60%, *t*(156) = 2.45, *p* < 0.05).

### Sporting ability

We investigated whether being an experienced athlete significantly moderated the prevalence of atypical footedness. Here, we first investigated the prevalence using a nonR–R classification. Sample type (general population vs. athletes) was extracted from 211 datasets (158 studies). The prevalence of non-right-footedness was found to be 22.80% (95% CI 20.20%, 25.40%) in the general population (179 datasets) and 25.50% (95% CI 20.20%, 30.70%) in experienced athletes (32 datasets). There was no evidence that these groups differed from another (*p* = 0.371).

**Figure 4:**
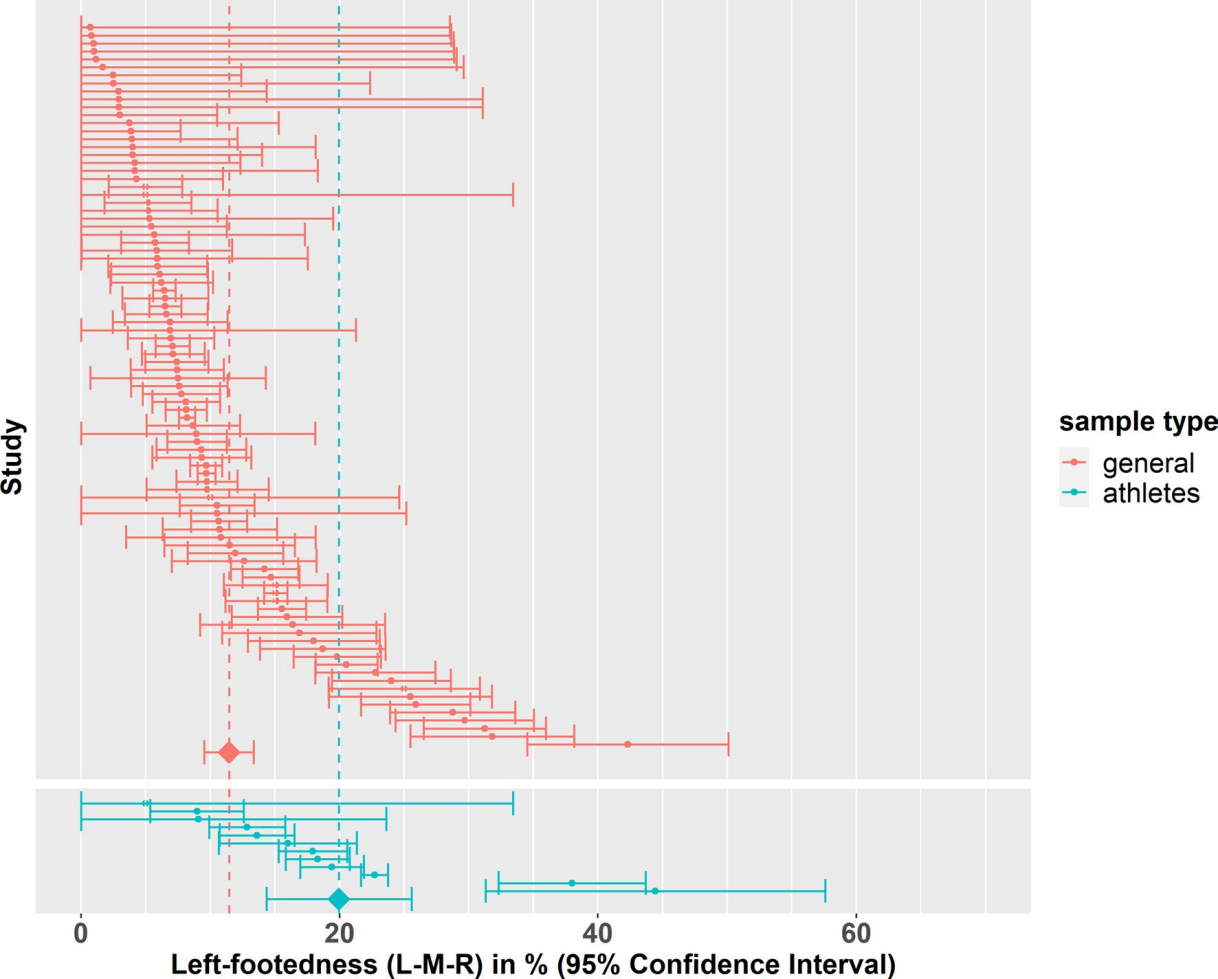
Forest plot for left-footedness (L-M-R) grouped by sample type (general population vs. experienced athletes). The 95% confidence interval for each study is represented by a horizontal line and the point estimate is represented by a dot. The dashed lines and diamonds indicate the point estimates for each group.

Since sporting ability has been claimed to selectively increase the rate of mixed-footedness^50^, we specifically investigated this moderator in the L-M-R classification system. Since only a subset of studies reported data in the L-M-R classification, only 103 datasets from 75 studies were included in the analysis. The prevalence of left-footedness (L-M-R) was found to be 11.40% (95% CI 9.53%, 13.40%) in the general population (91 datasets) and 20.00% (95% CI 14.34%, 25.60%) in athletes (12 datasets) (see Fig. 4). There was evidence that the prevalence of left-footedness (L-M-R) was higher in experienced athletes (+ 8.51%, SE = 2.97%, 95% CI = 2.59%, 14.40%, *t*(73) = 2.87, *p* < 0.01). No difference between experienced athletes and the general population could be detected for mixed-footedness however (*p* = 0.874).

### Age

**Figure 5:**
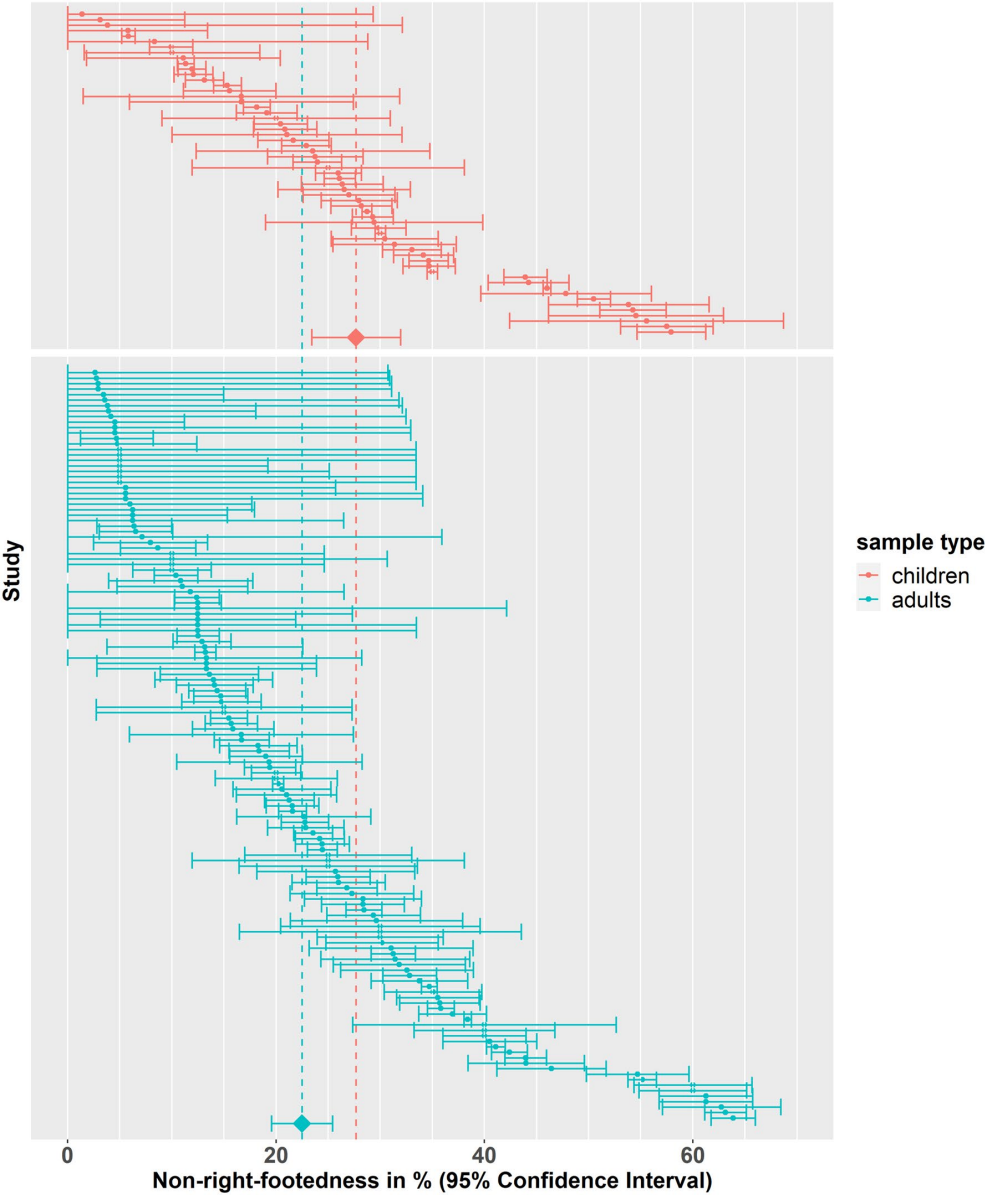
Forest plot for non-right-footedness grouped by sample type (children vs. adults). The 95% confidence interval for each study is represented by a horizontal line and the point estimate is represented by a dot. The dashed lines and diamonds indicate the point estimates for each group.

Age group was extracted from 196 datasets (150 studies). The prevalence of non-right-footedness was found to be 27.70% (95% CI 23.40%, 31.90%) in children’s samples (59 datasets) and 22.50% (95% CI 19.60%, 25.40%) in adult samples (137 datasets) (see Fig. 5). There was evidence that the prevalence of non-right-footedness was lower in adult samples (− 5.20%, SE = 2.61%, 95% CI = − 10.40%, − 0.03%, *t*(148) = − 1.99, *p* < 0.05). There was evidence of a handedness by age group interaction with left-handed adults being more often non-right-footed (+ 24.28%, SE = 10.04%, 95% CI = 3.95%, 44.60%, *t*(38) = 2.42, *p* < 0.05).

Since studies specifically reported increased mixed-footedness in children whereas left-footedness was on the same level as in adult cohorts, we repeated the previous analysis in studies using the L-M-R classification system. Age group (children or adult samples) was extracted from 100 datasets (75 studies) reporting the L-M-R classification. For left-footedness, the prevalence was found to be 9.63% (95% CI 6.99%, 12.30%) in children’s samples (32 datasets) and 13.79% (95% CI 11.35%, 16.20%) in adult samples (68 datasets) (see Fig. S2). There was evidence that the prevalence of left-footedness was higher in adult samples (+ 4.16%, SE = 1.80%, 95% CI = 0.58%, 7.75%, *t*(73) = 2.31, *p* < 0.05). For mixed-footedness, the prevalence was found to be 26.40% (95% CI 20.40%, 32.30%) in children’s samples and 18.10% (95% CI 14.40%, 21.80%) in adult samples (see Fig. S3). There was evidence that the prevalence of mixed-footedness was lower in adult samples (− 8.28%, SE = 3.51%, 95% CI = − 15.30%, − 1.28%, *t*(73) = − 2.36, *p* < 0.05).

An important variable that could mediate the difference between children and adults is the assessment of footedness. We therefore used the instrument for classification as a moderator for both subgroups (children and adults cohorts) to identify whether one group was specifically affected by the means of determining footedness. We found no differential effects of assessment instrument between the two groups in any classification system (nonR–R, L–R, L-M-R).

We then used the mean age as a meta-regressor to determine whether age influenced footedness as a continuous variable. The mean age of participants was reported in 139 datasets (108 studies). Meta-regression revealed no evidence of a moderating effect of mean age on the prevalence of non-right-footedness (*p* = 0.182). There was evidence for a handedness by age interaction with left-handers being more often non-right-footed with increasing age (+ 1.16%, SE = 0.47%, 95% CI = 0.19%, 2.12%, *t*(29) = 2.45, *p* < 0.05).

### Instrument

While we found no differences between children and adults regarding the influences of the instrument on prevalence of atypical footedness, this analysis did not provide insights about general effects of the chosen tool for assessing footedness, as potential influences on the prevalence of atypical footedness might simply have been identical in children and adults. We therefore used instrument as a moderator across all datasets. The type of instrument for footedness assessment was extracted from 194 datasets (145 studies). The prevalence of non-right-footedness in these datasets was found to be 21.80% (95% CI 16.60%, 26.90%) when assessed using the WFQ (62 datasets), 22.00% (95% CI 17.90%, 26.10%) when assessed using the LPI (22 datasets), 18.40% (95% CI 15.20%, 21.70%) when assessed as a preference to kick a ball (39 datasets), and 28.30% (95% CI 23.50%, 33.20%) when assessed using other preference inventories (71 datasets). There was no evidence in favour of a difference in the prevalence of non-right-footedness between instruments (all *p* > 0.05).

We repeated the analysis also in the L-M-R classification since the striking difference in the prevalence of mixed-footedness between the studies of Tran et al. and Coren^25,27^ might have been due to the instrument used to determine footedness. The type of instrument for footedness assessment was extracted from 99 datasets (73 studies) reporting the L-M-R classification. The prevalence of left-footedness (L-M-R) in these datasets was found to be 11.74% (95% CI 7.53%, 16.00%) when assessed using the WFQ (26 datasets), 9.68% (95% CI 6.89%, 12.50%) when assessed using the LPI (15 datasets), 12.11% (95% CI 8.99%, 15.20%) when assessed as a preference to kick a ball (15 datasets), and 13.19% (95% CI 9.55%, 16.80%) when assessed using other preference inventories (43 datasets). There was no evidence for a difference in the prevalence of left-footedness (L-M-R) between instruments (all *p* > 0.05).

**Figure 6:**
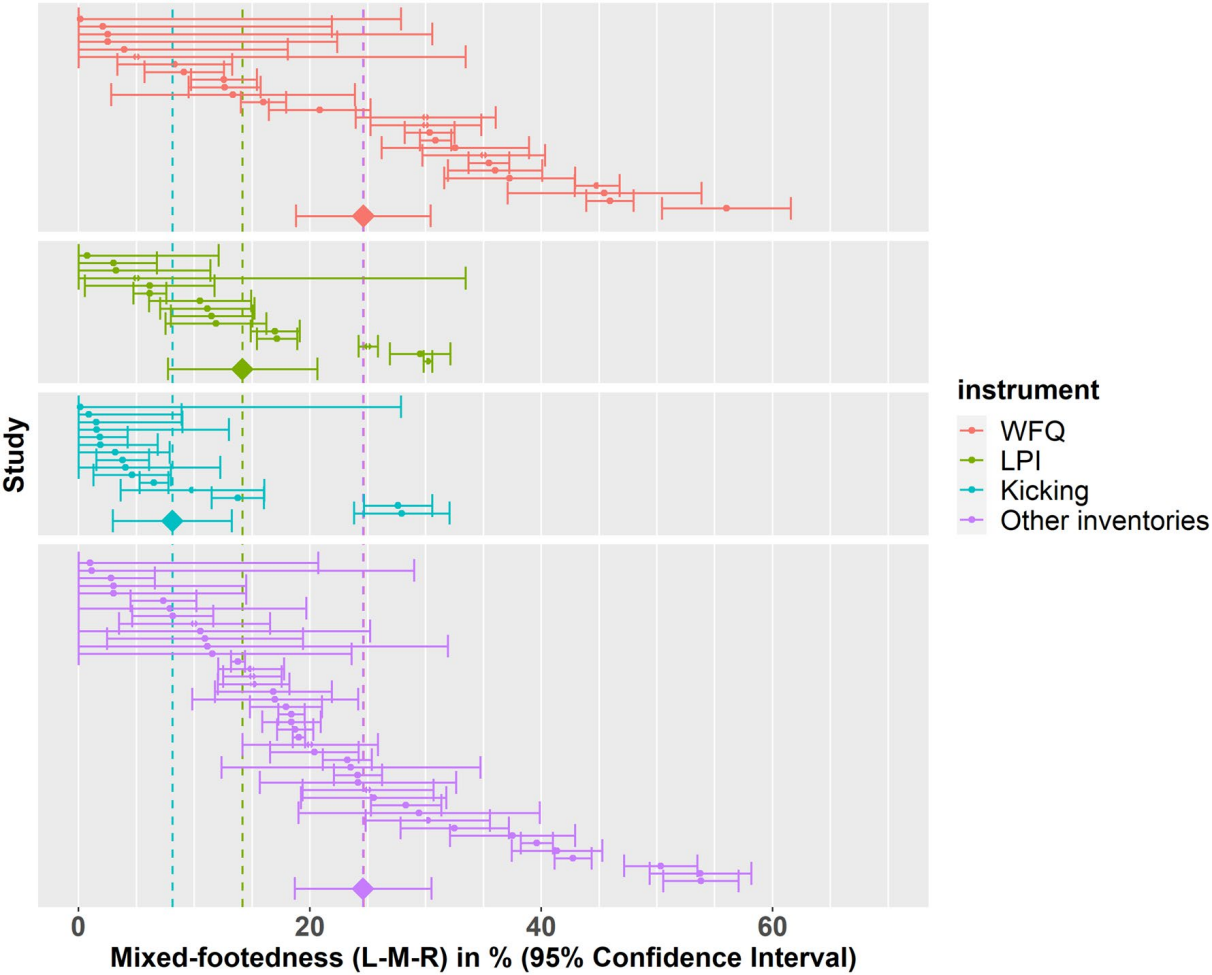
Forest plot for mixed-footedness (L-M-R) grouped by instrument used to assess footedness. The 95% confidence interval for each study is represented by a horizontal line and the point estimate is represented by a dot. The dashed lines and diamonds indicate the point estimates for each group.

The prevalence of mixed-footedness (L-M-R) in these datasets was found to be 24.60% (95% CI 18.77%, 30.40%) when assessed using the WFQ (26 datasets), 14.16% (95% CI 7.70%, 20.60%) when assessed using the LPI (15 datasets), 8.09% (95% CI 2.95%, 13.20%) when assessed as a preference to kick a ball (15 datasets), and 24.59% (95% CI 18.69%, 30.50%) when assessed using other preference inventories (43 datasets) (see Fig. 6). There was evidence for a lower prevalence of mixed-footedness (L-M-R) when footedness was assessed using the LPI (− 10.44%, SE = 4.36%, 95% CI = − 19.14%, − 1.73%, *t*(69) = − 2.39, *p* < 0.05) and kicking a ball (− 16.51%, SE = 3.90%, 95% CI = − 24.29%, − 8.74%, *t*(69) = − 4.24, *p* < 0.001) as compared to the WFQ. There was no evidence for a difference in the prevalence of mixed-footedness when assessed using other preference inventories compared to the WFQ (*p* = 0.997).

### Response format

The number of response options was extracted from 201 datasets (151 studies). The prevalence of non-right-footedness was found to be 22.80% (95% CI 18.80%, 26.80%) when participants were given two response options (73 datasets), 24.20% (95% CI 20.70%, 27.60%) when participants were given three response options (59 datasets), and 21.60% (95% CI 16.90%, 26.30%) when participants were given five response options (69 datasets). There was no evidence for a moderating effect of number of response options on the prevalence of non-right-footedness (all *p* > 0.05).

### Study location

Study location was extracted from 215 datasets (163 studies). The prevalence of non-right-footedness was 25.00% (95% CI 21.70%, 28.30%) when study participants were tested in European countries (94 datasets), 24.10% (95% CI 19.80%, 28.50%) when study participants were tested in American countries (63 datasets), 21.90% (95% CI 12.30%, 31.40%) when study participants were tested in African countries (6 datasets), 19.20% (95% CI 13.50%, 24.90%) when study participants were tested in Asian countries (40 datasets), and 26.70% (95% CI 18.30%, 35.10%) when study participants were tested in New Zealand or Australia (12 datasets). There was no evidence for a moderating effect of study location on non-right-footedness (all *p* > 0.05). However, interaction analysis with sex revealed a lower prevalence of non-right-footedness in female participants in Asian countries (− 16.89%, SE = 3.73%, 95% CI = − 24.34%, − 9.44%, *t*(64) = − 4.53, *p* < 0.001) compared to female participants in European countries.

### Publication year

Year of publication was extracted from 211 datasets (158 studies). Meta-regression revealed no evidence of a moderating effect of publication year on the prevalence of non-right-footedness (*p* = 0.304). There was, however, evidence in favor of a handedness by publication year interaction with mixed-handers being less often non-right-footed in early published studies (− 0.60%, SE = 0.28%, 95% CI = − 1.16%, − 0.04%, *t*(39) = − 2.15, *p* < 0.05). When year of publication was used as a categorical variable, the prevalence of non-right-footedness was found to be 22.40% (95% CI 4.43%, 40.30%) when studies were published before 1976 (5 datasets), 26.20% (95% CI 20.75%, 31.60%) when studies were published between 1976 and 1985 (15 datasets), 33.00% (95% CI 25.73%, 40.30%) when studies were published between 1986 and 1995 (19 datasets), 21.40% (95% CI 18.32%, 24.50%) when studies were published between 1996 and 2007 (70 datasets), and 21.20% (95% CI 17.71%, 24.60%) when studies were published between 2008 and 2019 (102 datasets). There was no evidence for a moderating effect of publication year on non-right-footedness (all *p* > 0.05).

## Discussion

In the present study, we report new data on footedness prevalence from a large epidemiology cohort and report the first meta-analyses on the prevalence of atypical footedness in the general population. Investigating 145,135 individuals from 164 studies revealed that the 95% CI for the point estimate of non-right-footedness ranges between 21.40% and 25.90%. This estimate is considerably higher than what has been determined for handedness as the 95% CI for non-right-handedness has been 13.90% and 22.30% in a recent meta-analysis^20^. If one assumes that handedness and footedness follow a similar distribution in the population, a possible reason for this higher point estimate could be attributed to the already mentioned social and cultural influences on handedness. Indeed, the meta-analysis by Papadatou-Pastou et al.^20^ found strong evidence that both East Asian and sub-Saharan African cohorts exhibit a significantly lower rate of left-handedness compared to Western societies. Our results, however, have only demonstrated minor influences of study location. There was also no effect of publication year supporting the idea that footedness might indeed be a “purer” measure of laterality in the population as has been suggested^19^. Interestingly, we found an interaction between publication year and handedness of the participants further indicating that the influence on footedness by year of publication in individual studies is mediated only by differences in handedness.

### Relationship between footedness and handedness

Using meta-analyses, we were able to illuminate the relationship between hand and foot preferences since we could provide accurate estimates across a large number of studies and participants. On top of the results from the meta-analyses, we also reported correlations from several large data sets in the ALSPAC cohort. Here, the correlations were around r = 0.5 indicating a moderate association between footedness and handedness. A potential reason why the association between these variables is not stronger could be explained by the overall smaller reliability of footedness measures ranging from 0.55 to 0.75 depending on the item^95^. Since variables cannot correlate more with another variable than with itself^96^, a generally lower correlation between handedness and footedness is to be expected. Using meta-analyses, we were able to corroborate previous findings that the co-occurrence between right-handedness and right-footedness is especially strong, whereas the association between left-handedness and left-footedness is less pronounced^97^. Our results show indeed that in an L-M-R classification system, 60.1% of left-handers were also estimated to be left-footed. However, only 3.2% of right-handers could be identified to be left-footed. Furthermore, we could find that mixed-handers are much more likely to be mixed-footed as well, a result in line with findings from Tran et al.^26^. However, in contrast to their results, we did not find an increased rate of left-footedness compared to right-footedness in mixed-handers indicating that mixed biases for limb preference are specifically associated.

### Sex differences in footedness

Sex differences in footedness have only rarely been investigated, but individual studies have indicated that males are more likely to exhibit left-footedness compared to females^98^. Regardless of classification, we also found robust sex differences in the prevalence of non-right/left-footedness. Here, males demonstrated increased rates of atypical, i.e. non-right- or left-footedness compared to females. This finding mimics sex differences that were found in handedness on the population level as males also demonstrate higher proportions of left-handedness compared to females^69,99^. Given these consistent findings between two distinct motor biases, the most pervasive explanation for this phenomenon seems to be a shared genetic basis underlying both limb preferences. To this day, no study has investigated the underlying genetic basis for footedness. However, research on the genetics of handedness is vast^57,73,100–104^. In contrast to early ideas of a single gene determining handedness^105^, modern genome-wide association studies (GWAS) could not find evidence of a single gene giving rise to hand use^106,107^. Recent studies investigating the molecular genetics of hand preference in over 300.000 individuals have successfully identified genes that play a crucial role in the determination of cellular pathways involved in neurodevelopment^57,104,108^. Nonetheless, there is still a wide gap in knowledge regarding the genetic basis of functional asymmetries. One possible reason for the inconsistencies in findings about the genetic foundation of handedness could be the strong nurture component in the ontogenesis of handedness^109^. Since our study found only a small moderating effect of culture, footedness seems to be the better candidate phenotype to understand the genetic basis of lateralization in motor behaviour and potentially cerebral asymmetries like processing of language and emotion^14,15^.

### Atypical footedness in clinical samples

The link between handedness and psychiatric and neurodevelopmental disorders has sparked high interest in the field of psychology and medicine alike resulting in a large variety of studies investigating this relationship^33,34,39,110^. In our analysis, we found a significant association between atypical footedness and clinical disorders indicating that handedness is not the only lateral bias that plays a distinguished role in this matter. Rather, it seems that there is a connection between clinical disorders and atypical motor biases in general. For example, the typically found left-sided cradling bias in the population^111^ is shifted to the right in clinical cohorts^112–114^. Our finding regarding the association between clinical conditions and footedness therefore highlights the importance of atypical motor asymmetries in psychological and neurological research. However, it remains elusive why that would be the case. It has been proposed that early-life stress might be the missing link between atypical development of both psychiatric and neurodevelopmental disorders and atypical functional hemispheric asymmetries including motor biases^115–118^. Here, early life stress could be a factor disrupting typical brain development ultimately causing altered functional as well as structural hemispheric asymmetries. Since there are currently no empirical studies directly investigating this link, we hope that our present results motivate more studies to look beyond the horizon of handedness as a candidate phenotype to help achieve a thorough understanding of the ontogenesis of psychopathologies.

### Footedness and sports

There is evidence of increased left-handedness in experienced athletes in handedness^20^. According to our analysis, this also holds true for footedness indicating that left-sidedness could indeed be advantageous in a variety of sports. Interestingly and contrary to our hypotheses, we could find no evidence that mixed-footedness is encountered more often in experienced athletes as has been suggested by Grouios et al.^50^. A possible reason for this phenomenon could be that for example professional football players, which comprised the large majority of the athlete data, still might have a clear preference to use one over the other even though they are usually more trained in using both feet^119^.

### Motor biases across the lifespan

Motor asymmetries seem to be present at a very early life stage, even prenatally in the case of handedness^120^. However, their stability across the lifespan remains an open question. For footedness, we found that the prevalence of non-right preference is actually increased in childhood compared to adulthood in line with results from for example Iteya et al.^121^. Interestingly, we were able to identify that the increase of non-right-footedness is exclusively due to higher rates of mixed-footedness in children as left-footedness was found to be higher in adults compared to children. Thus, our meta-analysis indicates that there is a clear shift towards more pronounced motor biases in either direction in adulthood and that brains become overall more asymmetrical during development. This result opposes predictions of the right hemi-aging model as this model solely predicts a shift to more right-sided motor biases^62^. An alternative explanation for this finding could be found in associations between motor biases and language lateralization. As language production and comprehension is strongly lateralized towards the left hemisphere whereas the right hemisphere specializes in prosody, intonation, and emotional speech^122^, the development of language skills throughout childhood and adolescence could significantly influence other cerebral asymmetries such as motor biases. Indeed, Szaflarski et al.^123^ found that there is an increase in language lateralization with increasing age in children and adolescents supporting this hypothesis.

### Limitations

A limitation of the present meta-analysis pertains to the general assessment of footedness in the population as it was almost exclusively assessed by a self-report rather than performing the actual task such as kicking the ball. Opposed to handedness, participants are usually less aware with which foot they prefer to perform a certain task from a footedness questionnaire as these activities are less prevalent in everyday life. While we do not deem it likely, the observed differences between the prevalence of handedness and footedness could at least to some extent relate to this issue.

Second, even though we used different classification systems in the meta-analysis, the underlying thresholds to determine for example an individual as left- or mixed-handed are entirely arbitrary and vary significantly from study to study. Unfortunately, this variable could not be integrated as a moderator as most studies did not report these thresholds given that the determination of footedness was largely not in the focus of the study. Given the very large study and sample size of the meta-analysis, effects of different thresholds will however likely be minor to the overall results.

A third limitation of this study concerns the analysis of clinical vs. healthy samples. Since studies investigating the prevalence of footedness from a single psychiatric or neurodevelopmental disorder were insufficient to conduct meaningful meta-analyses with, we subsumed all disorders into one clinically relevant category. While we found evidence in favour of the hypothesis that these disorders are associated with atypical footedness laterality as is the case for handedness, it remains unclear whether it is specific to a single disorder or a general phenomenon. We therefore urge researchers to assess this phenotype in future research on atypical lateralization patterns in clinical research to illuminate on this crucial issue.

Fourth, the comparison of athletes vs. the general population featured studies investigating football players in the large majority of the cases whereas only small subsets of studies investigated for example dancers, cyclists, or skiers. Thus, our obtained results might be specific for football players.

Finally, we were unable to investigate the interaction between sex and handedness as footedness was in the vast majority of cases reported separately for both variables. We therefore hope that researchers upload raw data files into online repositories to allow for more specific evaluations of raw data sets in the context of future meta-analyses.

## Conclusion

Overall, these results could speak in favour of a new perspective in laterality research. Future studies could investigate footedness as a viable candidate phenotype in the context of development and disorders. In that regard, it might be an even better phenotype compared to handedness, as footedness seems less prone to high variance in datasets due to cultural and social influences. It has to be emphasized however that while footedness might be better than handedness as a proxy for brain asymmetries, motor preferences in general remain rather poor predictors of overall cerebral lateralization given the low correlation between these variables^12,124,125^. While the sheer number of studies included in this meta-analysis already indicates the general interest in footedness across various scientific disciplines, there is still a big gap in knowledge regarding its genetic foundation. We hope that this meta-analysis inspires many fields of research to not only look at handedness, but also at footedness, a phenotype that is similarly easy to assess. Large datasets such as the UK biobank should thus also assess footedness in the future. Only if motor biases are investigated holistically might we one day understand their ontogenesis and their role in brain development.

## Supplementary information

Supplementary Figure S1

Supplementary Figure S2

Supplementary Figure S3

Supplementary Material

Supplementary Table 1

## Data Availability

All data and analysis codes are made available in the OSF project “Meta-analysis of human footedness” under the link: https://osf.io/n5rwh/?view_only=5d5a80ce8b264f30adc72acae2ff0e27.
