# Supplementary figures and images for: Four meta-analyses across 164 studies on atypical footedness prevalence and its relation to handedness
Source: Sci Rep. 2020 Sep 2;10:14501. doi: 10.1038/s41598-020-71478-w (PMC7468297; doi:10.1038/s41598-020-71478-w)

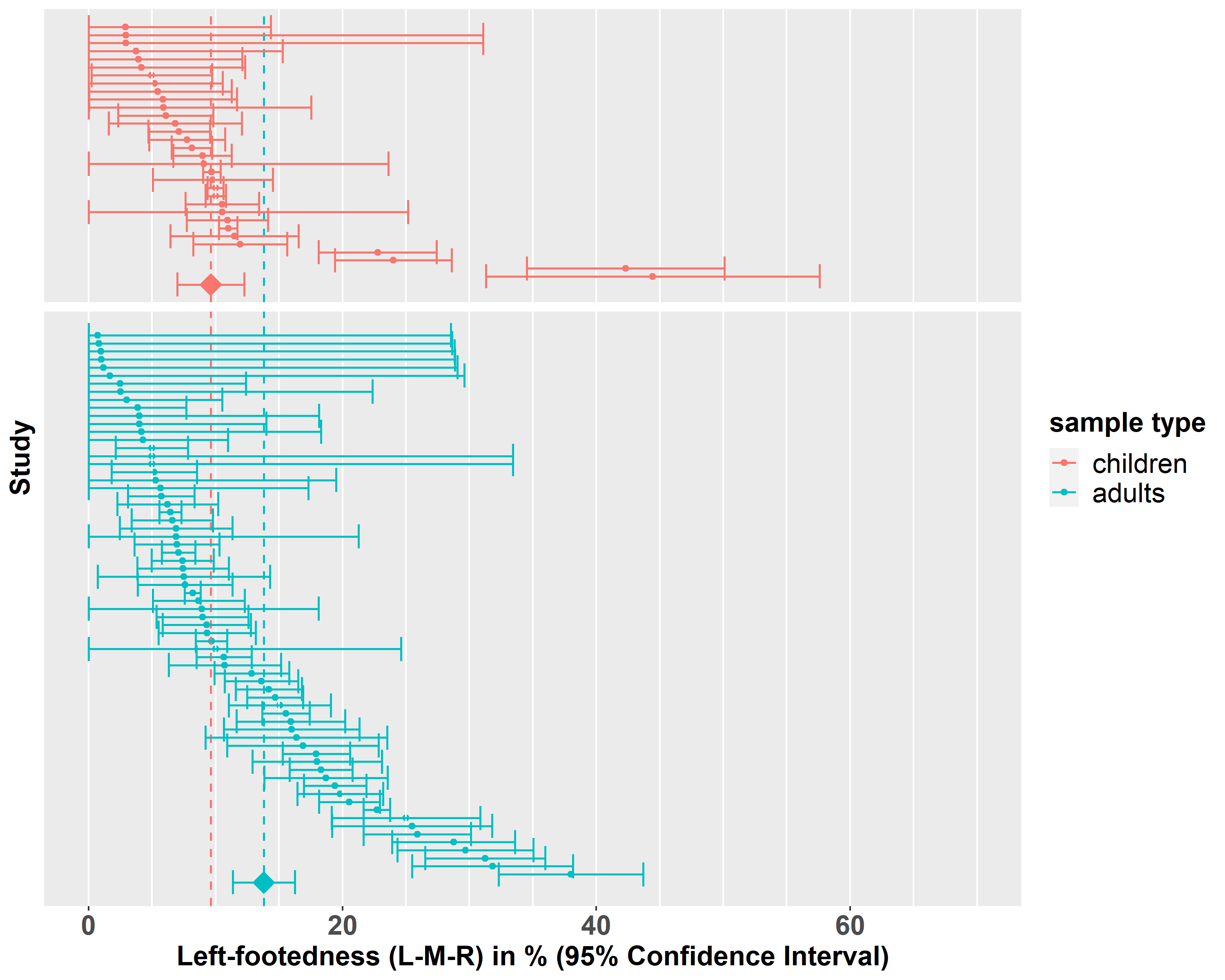

Supplement: Supplementary file 2 — Supplementary Figure S2 [file 41598_2020_71478_MOESM2_ESM.tiff]

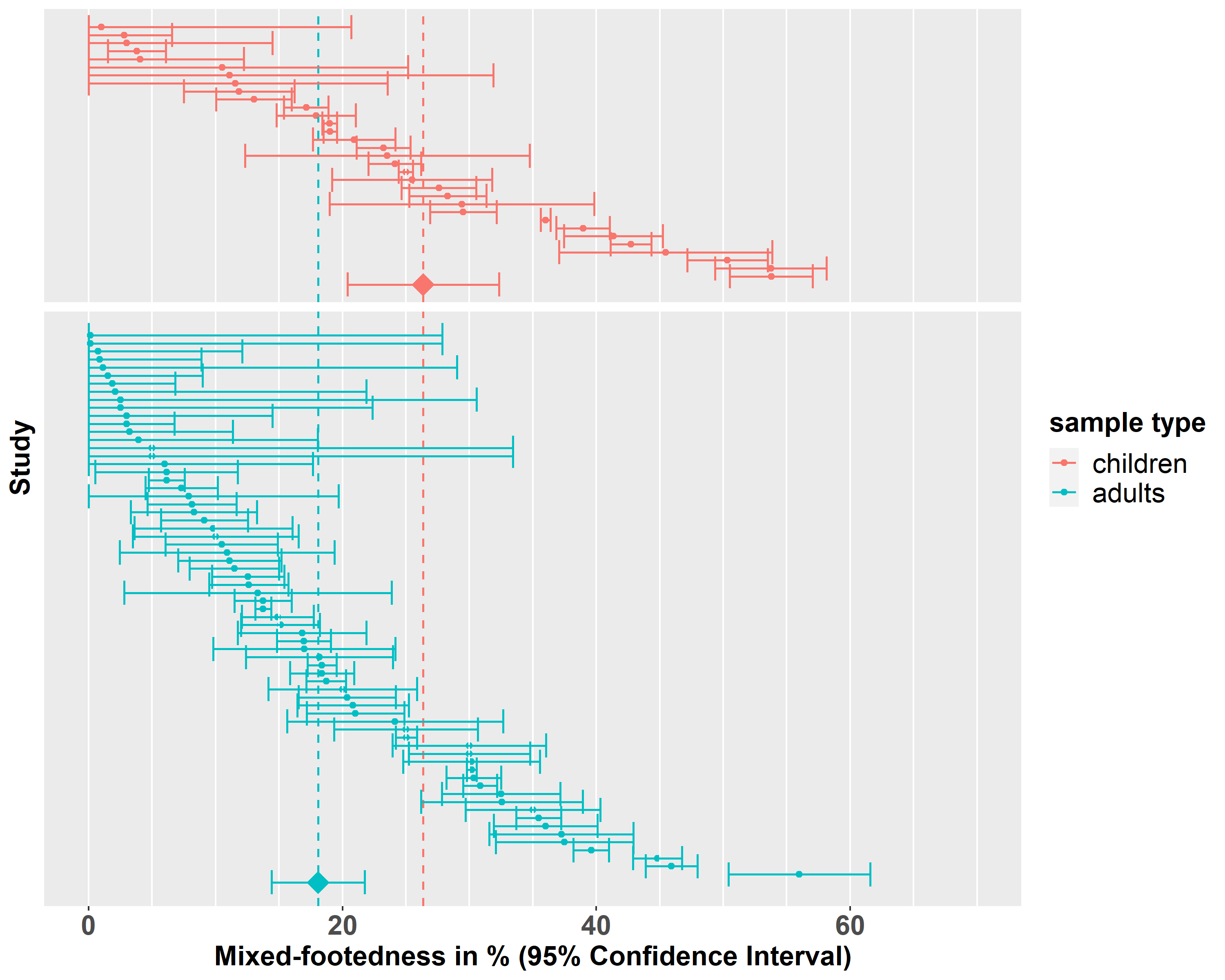

Supplement: Supplementary file 3 — Supplementary Figure S3 [file 41598_2020_71478_MOESM3_ESM.tiff]
