# Supplementary material for: Four meta-analyses across 164 studies on atypical footedness prevalence and its relation to handedness
Source: Sci Rep. 2020 Sep 2;10:14501. doi: 10.1038/s41598-020-71478-w (PMC7468297; doi:10.1038/s41598-020-71478-w)
Supplement: Supplementary file 4 — Supplementary Material [file 41598_2020_71478_MOESM4_ESM.docx]

**Four meta-analyses across 164 studies on atypical footedness prevalence and its relation to handedness**

Julian Packheiser^a^*, Judith Schmitz^b^, Gesa Berretz^a^, David P. Carey^c^, Silvia Paracchini^b^, Marietta Papadatou-Pastou^d^, Sebastian Ocklenburg^a,e^

^a^ *Institute of Cognitive Neuroscience, Biopsychology, Department of Psychology, Ruhr-University Bochum, Bochum, Germany*

*^b^ School of Medicine, University of St Andrews, St Andrews, UK*

*^c^ Perception, Action and Memory Research Group, School of Psychology, Bangor University, Bangor, UK*

^d^ School of Education, Department of Primary Education, National and Kapodistrian University of Athens, Athens, Greece

*^e^ Department of Psychology, University of Duisburg-Essen, Essen, Germany*
